# Integrated traditional Chinese and conventional medicine in the treatment of anemia due to lower-risk myelodysplastic syndrome: study protocol for a randomized placebo-controlled trial

**DOI:** 10.1186/s13063-021-05646-2

**Published:** 2021-10-18

**Authors:** Qin Zheng, Haitao Xu, Luxi Song, Zeying Yan, Manqin Sun, Xia Peng, Yiling Jiang, Ling Shi, Aiping Zhang, Zhihao Wu, Jiahui Lu, Meihong Luo

**Affiliations:** 1 Department of Hematology, Shanghai Baoshan Hospital of Integrated Traditional Chinese and Western Medicine (Baoshan Hospital Affiliated to Shanghai University of Traditional Chinese Medicine), Shanghai, 201999 China; 2 Department of Hematology, Shanghai Municipal Hospital of Traditional Chinese Medicine, Shanghai University of Traditional Chinese Medicine, Shanghai, 200071 China; 3 Department of Hematology, Shanghai Jiao Tong University Affiliated Sixth People’s Hospital, 200233 Shanghai, China; 4 Department of Hematology, Rui Jin Hospital Affiliated to Shanghai Jiao Tong University School of Medicine, 200020 Shanghai, China

**Keywords:** Anemia of lower-risk myelodysplastic syndrome, Traditional Chinese medicine, Treatment, Clinical trials

## Abstract

**Background:**

Erythropoiesis and iron homeostasis are closely related; anemia due to lower-risk myelodysplastic syndromes (MDS) remains difficult to treat. In the last decade, we have been committed to improving the regulation of iron metabolism using traditional Chinese medicine (TCM). Previous studies have found that the TCM Yi Gong San (YGS) can reduce the expression of transferrin by inhibiting hepcidin overexpression caused by inflammation, promote the outward transfer of intracellular iron, and improve the symptoms of anemia. Here, our study aimed to compare the efficacy of a conventional drug with YGS with that of conventional medicine with placebo to provide a scientific basis for making clinical decisions.

**Methods:**

A prospective, multicenter, double-blinded, randomized controlled clinical trial will be conducted to evaluate the therapeutic efficacy of conventional medicine combined with YGS with that of conventional medicine alone in the treatment of MDS. A total of 60 patients would be enrolled in this study, with each treatment group (conventional medicine + YGS and conventional medicine + placebo) comprising 30 patients. Oral medication would be administered twice daily for 3 months. All patients would be followed up throughout the 3-month period. The primary outcome was measured by assessing blood hemoglobin level. The secondary outcome was measured by assessing TCM symptom score, iron metabolism, hepcidin levels, and inflammatory factors.

**Discussion:**

This trial would aim to demonstrate the effectiveness and feasibility of YGS in the treatment of lower-risk MDS anemia, as well as its impact on inflammatory factors and iron metabolism in patients with lower-risk MDS.

**Trial registration:**

Chinese Clinical Trials Registry (http://www.chictr.org.cn/) ChiCTR1900026774.  Registered on October 21, 2019.

## Background

Myelodysplastic syndromes (MDS) are a heterogeneous group of hematologic neoplasms characterized as a clonal disorder of the hematopoietic stem/progenitor cells leading to a host of hematological malignancies. MDS can be manifested as one or more morbid hematopoiesis of the bone marrow hematopoietic cells, ineffective hematopoiesis, and high-risk acute myeloid leukemia transformation [1]. Anemia is the most common clinical manifestation in patients with MDS. Approximately 60%–80% of patients have symptoms of anemia, and nearly 50% of MDS patients have severe anemia with an increased risk for heart and lung failure. Patients with International Prognostic Scoring System-Revised score of ≤3.5 has been categorized as lower-risk MDS subpopulation according to the 2012 International Working Group criteria. According to the European MDS statistics, lower-risk MDS accounts for approximately 70% of the entire MDS population, and most lower-risk MDS patients have symptomatic anemia; therefore, improving anemia and alleviating the symptoms of anemia are the main treatment goals for patients with lower-risk MDS [2].

The clinical treatment of lower-risk MDS anemia has always been a challenging task, despite the development of various treatment methods in Western medicine, including erythropoietin, immunosuppressants (cyclosporine A), immunomodulators (lenalidomide amine and thalidomide), and those undergoing clinical trials. A previous study reported that in patients with MDS anemia treated with the aforementioned drugs, 80–90% still require blood transfusion, and blood transfusion dependence (1 U red blood cell transfusion at least every 8 weeks within 4 months) is negatively related to patient survival [3].

Lower-risk MDS anemia, also known as ineffective hematopoiesis, is primarily caused by the excessive proliferation of red blood cell precursors, which fail to mature and impair the ability of red blood cells to carry oxygen in the blood. Additionally, erythropoiesis and iron homeostasis are closely related. Abnormal iron metabolism is an independent risk factor affecting the prognosis of patients with lower-risk MDS [2], and hepcidin plays a key role in iron homeostasis. MDS has a complex iron regulation mechanism: anemia, hypoxia, inflammation, and iron overload, which have opposite effects on hepcidin production. Compared with healthy individuals, patients with MDS deprived of blood transfusion have significantly increased serum ferritin (SF) and hepcidin levels, but have decreased hepcidin/SF ratio [4, 5]. In patients with lower-risk MDS undergoing blood transfusion, the hepcidin levels initially increase; however, as the amount of transfused blood increases, the hepcidin levels gradually decrease, eventually causing iron overload [6]. Iron overload would further aggravate the underlying hematopoiesis deficiency in those with MDS, making blood transfusion ineffective [2].

In the last decade, we have been committed to improving the mechanism of iron metabolism using traditional Chinese medicine (TCM). According to the TCM theory, spleen deficiency is an important triggering factor of MDS, and the approach called “tonifying the spleen” in TCM clinical practice is a crucial step of MDS treatment. The spleen is responsible for maintaining the normal distribution of iron in the body. The implementation of this function depends on the transportation of spleen *Qi*. Therefore, the deficiency of spleen *Qi* and the diminished function of spleen would cause an iron distribution disorder. Yi Gong San (YGS) is a representative TCM prescription for promoting the movement of spleen *Qi*. Previous studies have found that YGS used in TCM has no effect on iron metabolism in normal mice. However, it can reduce the expression of transferrin by inhibiting hepcidin overexpression caused by inflammation, promoting the outward transfer of intracellular iron and thus improving anemia [7–9]. Reports from many clinical trials have shown that YGS can improve inflammation-induced anemia (anemia due to chronic diseases) [10]. In fact, prior to blood transfusion, lower-risk MDS have the same pathological process of iron metabolism with anemia due to chronic diseases. However, it is not clear whether YGS is a suitable therapeutic candidate for anemia in patients with lower-risk MDS. Thus, this study aimed to compare the efficacy of conventional drug with YGS with that of conventional drug alone, as a basis for making clinical decisions.

## Methods

### Objective and design

This multicenter, pragmatic, randomized, and controlled trial is conducted to evaluate the effectiveness of integrating YGS into the conventional treatment for anemia in patients with lower-risk MDS. A total of 60 patients would be recruited and randomly assigned to one of the two treatment groups. During the treatment period, patients in both arms would be followed up for 3 months. The primary outcome (hemoglobin) and secondary outcome measures (TCM symptom score, iron metabolism, hepcidin levels, and inflammatory factors) would be assessed at different points within the trial period.

The study has been approved by the research ethics committee of the Shanghai Baoshan Hospital of Integrated Traditional Chinese and Western Medicine (#201809-01). This trial adhered to the Declaration of Helsinki and has been registered at the Chinese Clinical Trials Registry (ChiCTR1900026774) on October 21, 2019. The trial results would be reported according to the Standard Protocol Items: Recommendations for Interventional Trials statement and the latest version of the Consolidated Standards of Reporting Trials statement.

### Study setting and participants

All adult patients with lower-risk MDS admitted at the respiratory departments in three tertiary care hospitals would be screened and enrolled. Eligible patients would be asked to provide an informed written consent and would be centrally randomized to either the integrated YGS and conventional medicine group or the conventional medicine group. For both groups, all medications would be administered orally for 3 months (12 weeks). Patient recruitment has begun in March 2020 and should be completed in December 2021.

The inclusion criteria are as follows:

- Patients aged > 18 years and older
- Patients whose peripheral blood cell, bone marrow smear, gene, and chromosome profiles meet the criteria for lower-risk MDS according to the 2020 NCCN guidelines (V1)
- Patients with a hemoglobin level of < 110 g/L
- Patients whose symptoms meet the spleen *Qi* deficiency pattern of MDS according to the Standard Criteria for Syndrome Differentiation by TCM[11]

The exclusion criteria are as follows:

- Patients who are blood transfusion dependent
- Pregnant or lactating women, and women planning to become pregnant (must undergo urine pregnancy test, a standard test for women of childbearing age, before treatment)
- Patients with liver and kidney function insufficiency (whose blood aspartate aminotransferase, alanine aminotransferase, or creatinine concentrations exceed the normal value by more than 3 times the upper limit)
- Patients who recently participated in clinical trials of other drugs (within 2 weeks of TCM, within 7 half-lives of Western medicine)
- Patients with comorbidities that may cause anemia such as neoplastic diseases
- Patients who are unwilling to cooperate
- Patients who cannot complete the specified observation items after being selected

### Randomization and double-blind approach

**Fig. 1:**
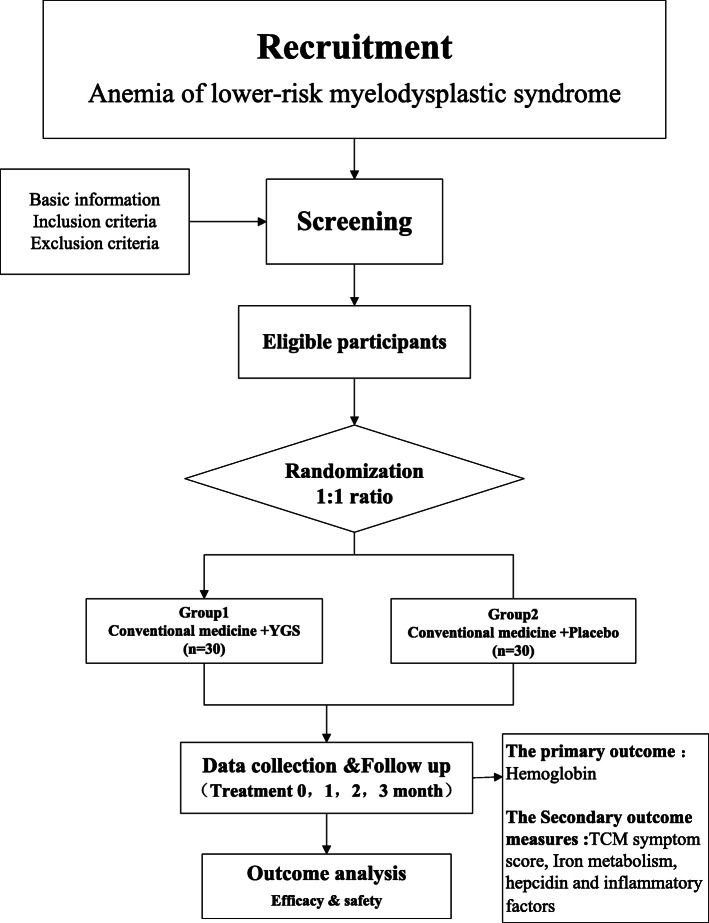
Flow chart of the study design

Participants who met the inclusion criteria and signed an informed consent form would be allocated to a group (Fig. 1). Based on a stratified block randomization design with a 1:1 ratio, two groups of patients would be assigned by the study center at the time of study. Allocation of patients is based on the random numbers (1 to 60) generated using an SPSS software version 25.0, and patient’s details are saved and kept in a sealed envelope by an independent clinical statistician based on the double-blind research method. In the study, the procedures of generating random numbers, evaluating the results of primary and secondary outcomes, and statistical analysis are independently performed by dedicated personnel who are blinded to outcome data. Upon completion of the study and data analysis, the efficacy and safety of YGS treatment shall be evaluated. During the clinical trial period, the treatment regimen of both study groups would be managed by their respective study center/hospital according to the approved treatment plan. If a patient develops a serious adverse reaction or life-threatening condition during the course of clinical trial, the clinician responsible for the patient must provide the best on-site medical care. Simultaneously, the clinician must immediately report the incidence to the inspector, upon which the letter containing patient’s treatment grouping information needs to be opened to break the blind, and the type of drug used is identified to ensure that the patient is provided with the appropriate emergency medical assistance. Once a blind is uncovered, the case would be regarded as a shedding case and would be excluded from the statistics, and any occurrence of adverse reactions would be recorded.

### Intervention treatment

All patients would be treated with conventional medicine according to the MDS 2020 NCCN guidelines (V1) and TCM guidelines for the diagnosis and treatment of MDS (2019) [12]. For the conventional medicine and YGS treatment, patients would be given herbal interventions based on the spleen *Qi* deficiency pattern of MDS. YGS is an oral decoction containing five herbs: Ginseng Radix et Rhizoma (*Ren-Shen*), Glycyrrhizae Radix et Rhizoma (*Gan-Cao*), Citri Reticulatae Pericarpium (*Chen-Pi*), Atractylodis Macrocephalae Rhizoma (*Bai-Zhu*), and Poria Sclerotium (*Fu-Ling*). Granules containing these herbs are manufactured by Jiangyin Tianjiang Pharmaceutical Co., Ltd with strict compliance to Good Clinical Practice conditions in terms of the production process and packaging of mixed granules for TCM. Each YGS granule contains 20 g of each herbal raw material (Table 1).

**Table 1:** Weight and composition of YGS and placebo granules

Chinese name (*pinyin*)	Latin name	Granule weight (g)	Herbal raw material weight (g)
Yi Gong San granules		**44.8**	
*Ren-Shen*	Ginseng Radix et Rhizoma	6.7	20
*Bai-Zhu*	Atractylodis Macrocephalae Rhizoma	14.7	20
*Fu-Ling*	Poria Sclerotium	6.7	20
*Chen-Pi*	Citri Reticulatae Pericarpium	8.2	20
*Gan-Cao*	Glycyrrhizae Radixet Rhizoma	8.5	20
Placebo granules		**44.8**	0

For the conventional medicine group, patients would be administered placebos containing maltodextrin, lactose, and food coloring, according to the modern pharmaceutical technology and placebo specification for granules. The placebos appear identical to YGS granules in terms of packaging, weight, odor, and color. In all patients, the medications would be administered twice daily, after breakfast and dinner, and up to 3 months (12 weeks).

### Data collection

Most patients in the outpatient clinic would be enrolled in the study. We would first obtain the patient’s informed consent. Then, we will collect the patient’s demographic data (sex, age, occupation, home address, diagnosis, and past history of illness) and help the patient fill out the TCM symptom scale to determine the type of symptom presented. A peripheral blood sample would be collected from each included patient prior to the initiation of treatment. The blood sample would be examined for various biochemical parameters such as liver and kidney function, iron metabolism-related indicators, hepcidin levels, and inflammatory factor-related markers. By randomly assigning them into either group, the patients would be administered with oral doses of conventional medicine with YGS or placebo for 3 months (12 weeks). At 1-, 2-, and 3-month intervals, the patients will be followed up in order to determine who among them would be suitable to fill up a new TCM symptom scale form to rate their symptoms, and a fresh peripheral blood samples would be collected (Table 2). Use case record form to collect patient clinical data and test data, and enter the information and data into electronic data capture.

**Table 2:**
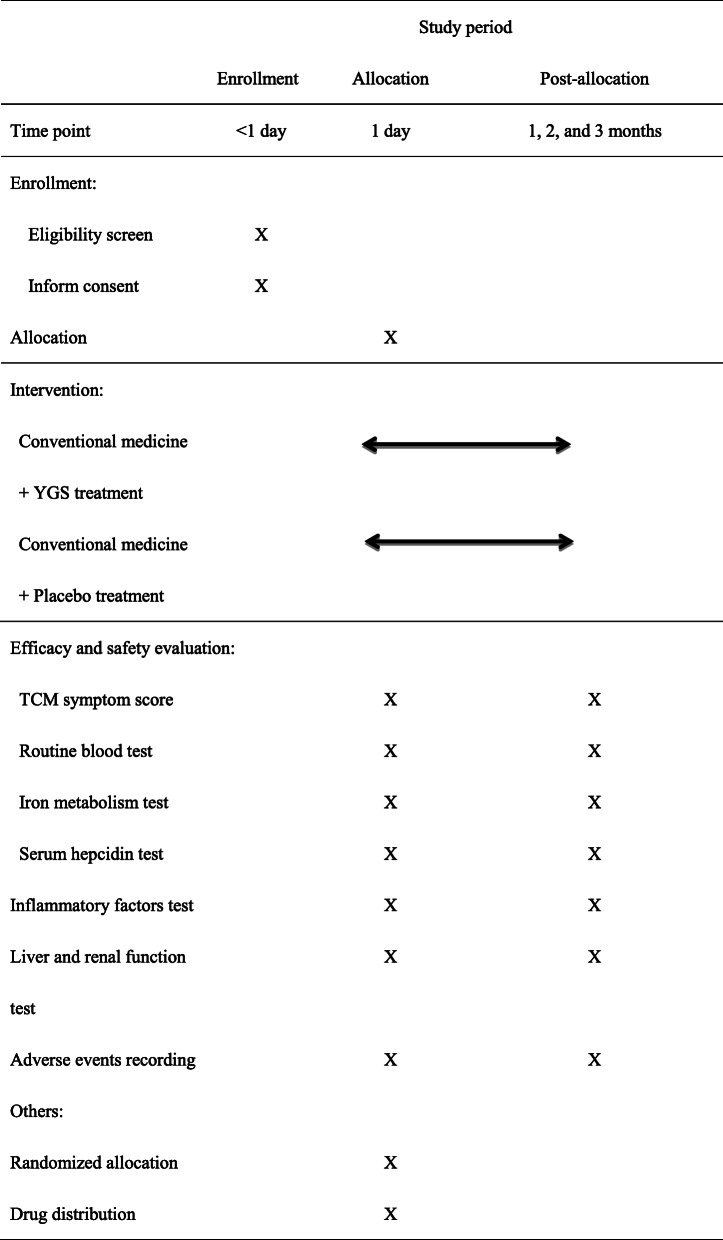
Study schedule for patients

### Efficacy assessment

#### Primary outcome

The primary outcome is the blood hemoglobin level; by comparing it to the baseline level prior to the initiation of treatment, an increase of 10 g/L is considered as an effective treatment outcome.

#### Secondary outcomes

The secondary outcomes measure the changes in TCM symptom scores, iron metabolism, hepcidin levels, and inflammatory factors. Spleen *Qi* deficiency syndrome is primarily characterized by poor appetite, fatigue, abdominal distension after meal or in the afternoon, and abnormal stool. The effectiveness of TCM is evaluated using the TCM syndrome score method (Table 3). According to the Standard Criteria for Syndrome Differentiation by TCM [11], a reduction in TCM syndrome score by ≥30% indicates that the clinical symptoms of spleen *Qi* deficiency either improved or disappeared and the treatment is considered clinically effective. The TCM syndrome score is calculated as follows: [(scores before treatment − scores after treatment) ÷ scores before treatment] × 100%. The indicators for iron metabolism are serum iron, total iron-binding capacity, SF, and soluble transferrin receptor. Inflammatory factors include interleukin (IL)-1, IL-6, and tumor necrosis factor (TNF)-α.

**Table 3:** TCM syndrome scores for spleen *Qi* deficiency

Symptom	Score		
	Mild = 2	Moderate = 4	Severe = 6
Reduced food intake	No appetite, but maintain the original intake	Intake volume reduced by 1/3 compared before the onset of symptoms	Intake volume reduced by 2/3 compared before the onset of symptoms
Fatigue	Slightly tired and inactive but able to perform basic daily activities	Obvious sign of burnout, barely performing daily activities	Systemic weakness of the limbs, unable to perform daily activities
Bloating after meal	Slight abdominal distension, which can disappear within half an hour; is not severe; and does not require medications	Slight abdominal distension, which can disappear within mild; is not severe; and does not require medications	Abdominal distension cannot be improved within 2 h, affecting life, or taking ineffective medicines
Abnormal stool	Soft stools or slightly watery stools, piles are not formed, frequency of bowel movements: 2–3 times/day	Watery stools, loose stools, frequency of bowel movements: 4–5 times/day or 1–2 times/day	Loose stools, more than 3 times/day
Reduced talkativeness/responsiveness	Low energy, not interested to talk or respond	Mental fatigue, sleepy, not interested to engage in conversation	Extremely fatigued, idle
Absence of taste/ thirst	Occasional tasteless	Sometimes tasteless, reduced water intake	Persistent absence of taste, not thirsty all day long
Abdominal discomfort	Occasional or faint abdominal pain	Abdominal pain often occurs, lasting no more than 2 h	Abdominal pain is more obvious, lasting more than 2 h, or persistent
Nausea and vomiting	Occasionally nausea	Sometimes nausea, occasionally vomiting	Frequent nausea and vomiting
Excessive bowel gas	Occasional	Often	Hyperactive
Yellowish complexion	Yellowish complexion and reduced luster	Yellowish complexion and much reduced luster	Yellowish complexion and dull
Edema	Mild edema of the face or lower limbs	Edema of the face and limbs	Obvious body edema
Difficulty in Defecation	Straining to defecate	Require great effort to defecate	Hard to defecate or severely constipated

No symptom = 0

### Adverse event reporting

Details of the mild or severe adverse effects following TCM treatment would be recorded in the “case report form,” which comprise the time of occurrence, clinical manifestations, number of treatment elapsed days, duration, outcome, and possible side effects of the drug. Patients with abnormal laboratory test results must be followed up until the test results return to normal or to the level prior to the administration of medications; the clinicians shall determine whether the abnormalities are related to the treatment drug. If a serious adverse reaction develops, the “serious adverse event form” should be filled out, and the incident would be reported to the sponsor, research ethics committee, the Safety Supervision Department of National Medical Products Administration, and the Health Administration Department within 24 h. The sponsor and research ethics committee in charge will conduct the quality control and patient compliance assessment in this trial.

### Sample size considerations

Based on the sample size estimation formula (*n*1= *n*2 =2×[(μα+μβ)σ/δ]^2^+ μα^2^), the main research outcome indicator is the change in hemoglobin (Hb) levels, which will be used to estimate the sample size; compared with the baseline results, the Hb level of the experimental group (conventional medicine + YGS) increased to 20 g/L, while the Hb level of the control group (conventional medicine + placebo) increased to 14 g/L (standard deviation: 7 g/L, *n*1=group 1, *n*2= group 2, σ=14, δ=7, bilateral α=0.05, μα=1.96, unilateral β=0.2, μβ=0.84); therefore, each group should comprise 22 patients; considering a 30% loss due to follow-up, each group should comprise 30 patients. Therefore, we intend to recruit 60 patients from March 2020 to December 2021.

### Statistical analyses

A descriptive statistical analysis of all the quantified variables in this study would be performed. The mean, median, and standard deviation will be calculated for quantitative variables, while the absolute and relative frequencies will be calculated for qualitative variables. The SPSS software (version 25.0; IBM Inc., USA) would be used for data processing and analysis. Statistical analysis would be based on the intention-to-treat and per-protocol population principles. The missing data can be processed using the last observation carried forward method. To compare the changes in TCM symptom scores and hematological data of patients before and after taking intervention treatment, multiple comparisons would be performed. A repeat measurement data analysis of variance will be initially conducted followed by a separate effect analysis, including comparisons between groups at each time point, using a multivariate logistic regression model to analyze the correlation between iron metabolism, hepcidin levels, and IL-6, TNF-α, IL-1 expression changes, and curative effect. Additionally, a subgroup analysis can be performed according to the R-IPI, and covariance analysis can be performed considering the factors such as age and sex. A two-sided *P* value of < 0.05 is considered significant.

## Discussion

The release of inflammatory factors plays an important role in the development of lower-risk MDS, including the upregulation of cytotoxic T cells [13]. Bone marrow fibroblasts and macrophages of patients with MDS continuously release IL-6, IL-1, TNFs, and other inflammatory factors to further aggravate the compromised hematopoiesis in the bone marrow and promote the apoptosis of bone marrow cells [14]. Patients with MDS often have a low immune function and increased co-infection risk especially those with leukopenia. Co-infection would further stimulate the release of inflammatory factors in the body, especially IL-6 and IL-1, that can induce liver hepcidin overexpression and inhibit iron absorption and release of stored iron [15], resulting in iron metabolism disorders that are similar to anemia induced by chronic diseases. C-reactive protein (CRP) levels indirectly reflect the expression of inflammatory factors. Previous studies have shown that serum CRP levels in patients with MDS are positively correlated with the hepcidin, especially the RAEB type [4]. Similar to SF, CRP has a significant effect on patient’s survival, indirectly indicating that inflammatory factors can affect the survival of patients with MDS [2].

YGS is an oral concoction first reported in the Pediatric Medicine Card Straight Tactic, a famous classical book of TCM written approximately 900 years ago. YGS is known for its functions of tonifying the splenic *Qi* and gasification stagnation. YGS has been traditionally used in Korea to treat a variety of inflammatory diseases; pretreatment with YGS inhibited the production of TNF-α훼 and IL-6 in LPS-stimulated mouse peritoneal macrophages [16], which is consistent with our previous animal experiment results [7], indicating the confirmed inhibitory effect of YGS on inflammatory factors. In our current study, we intend to enroll patients with MDS anemia who are not dependent on blood transfusion and provide them with relevant treatments during the early stages of lower-risk MDS. In patients with lower-risk MDS, early T cell function is increased, and inflammatory factors are highly expressed [17], which is the same as the pathological process of iron metabolism in ACD. Therefore, based on a previous study, we hypothesized that YGS can regulate iron metabolism by reducing the inflammatory factors of lower-risk MDS, thereby improving anemia. We intend to include 60 patients with lower-risk MDS anemia; conduct a randomized, double-blind, placebo-controlled parallel trial; administer oral YGS granules or placebo granules to included patients; and evaluate the clinical efficacy of this treatment.

This trial has the following advantages: (1) This study is a randomized, double-blind, placebo-controlled, multicenter study that is scientific and rigorous. (2) At present, no relevant research has evaluated the effects of TCM on inflammatory factors and iron metabolism in MDS. The findings of this study would fill this gap of knowledge in the literature. This study has certain limitations. It only evaluates the early stages of MDS; most of the patients with MDS are already on the late stages when they see a doctor. Therefore, it might be difficult to recruit patients, and we have to set the sample size based on the minimum number of cases required for statistical analyses. In this study, MDS treatment using Western medicine is also recorded as an individualized treatment regimen, which would be further analyzed as a stratified group of results in our statistical analyses.

The results of this clinical trial would reveal the effectiveness and feasibility of YGS in the treatment of lower-risk MDS anemia, as well as its impact on inflammatory factors and iron metabolism in patients with lower-risk MDS. We hope that the combination treatment of Chinese and Western medicine can delay the progression of MDS and improve the prognosis and survival of patients.

## Trial status

This study is currently in the process of recruiting participants. The protocol version number is 2.0, dated October 08, 2018. Recruitment of participants commenced on March 23, 2020, and is expected to end on December 31, 2021. At the time of manuscript submission, we have recruited 19 patients from three hospitals concurrently; therefore, this clinical trial is expected to be completed on time.

## Data Availability

The datasets generated and/or analyzed during the current study are not publicly available, owing to the protection of privacy for patients, but they are available from the corresponding author upon reasonable request.

## Abbreviations

MDS: Myelodysplastic syndromes
TCM: Traditional Chinese medicine
YGS: Yi Gong San
ChiCTR: Chinese clinical trials registry
SF: Serum ferritin
IL-1: Interleukin-1
IL-6: Interleukin-6
TNF-α: Tumor necrosis factor-α
CRP: C-reactive protein
